# The phosphohistidine phosphatase SixA dephosphorylates the phosphocarrier NPr

**DOI:** 10.1074/jbc.RA120.015121

**Published:** 2020-11-23

**Authors:** Jane E. Schulte, Manuela Roggiani, Hui Shi, Jun Zhu, Mark Goulian

**Affiliations:** 1 Graduate Group in Biochemistry and Molecular Biophysics, Perelman School of Medicine, University of Pennsylvania, Philadelphia, Pennsylvania, USA; 2 Department of Biology, University of Pennsylvania, Philadelphia, Pennsylvania, USA; 3 College of Food Science, Southwest University, Beibei, Chongqing, China; 4 Department of Microbiology, Perelman School of Medicine, University of Pennsylvania, Philadelphia, Pennsylvania, USA; 5 Department of Physics & Astronomy, University of Pennsylvania, Philadelphia, Pennsylvania, USA

**Keywords:** histidine, bacterial protein phosphatase, protein phosphorylation, phosphoryl transfer, *Escherichia coli*, phosphoramidate hydrolase, phosphotransferase system, cross talk, π-pHis, π-phosphohistidine, τ-pHis, τ-phosphohistidine, B-PER, bacterial protein extraction reagent, CFU, colony forming units, ECL, enhanced chemiluminescence, FRT, FLP recombination target, (His)_6_ or (His)_8_, polyhistidine, IPTG, isopropyl-β-D-thiogalactopyranoside, NPr-P, phosphorylated NPr, P_*trc*_, *trc* promoter, PEP, phosphoenolpyruvate, SixA-P, phosphorylated SixA, TBST, Tris-buffered saline and Tween solution, TEV, tobacco etch virus

## Abstract

Histidine phosphorylation is a posttranslational modification that alters protein function and also serves as an intermediate of phosphoryl transfer. Although phosphohistidine is relatively unstable, enzymatic dephosphorylation of this residue is apparently needed in some contexts, since both prokaryotic and eukaryotic phosphohistidine phosphatases have been reported. Here we identify the mechanism by which a bacterial phosphohistidine phosphatase dephosphorylates the nitrogen-related phosphotransferase system, a broadly conserved bacterial pathway that controls diverse metabolic processes. We show that the phosphatase SixA dephosphorylates the phosphocarrier protein NPr and that the reaction proceeds through phosphoryl transfer from a histidine on NPr to a histidine on SixA. In addition, we show that *Escherichia coli* lacking SixA are outcompeted by wild-type *E. coli* in the context of commensal colonization of the mouse intestine. Notably, this colonization defect requires NPr and is distinct from a previously identified *in vitro* growth defect associated with dysregulation of the nitrogen-related phosphotransferase system. The widespread conservation of SixA, and its coincidence with the phosphotransferase system studied here, suggests that this dephosphorylation mechanism may be conserved in other bacteria.

Histidine phosphorylation occurs in numerous metabolic and signal transduction pathways. In many cases, phosphohistidine appears in enzyme intermediates of phosphoryl transfer reactions between proteins or small molecules (1, 2, 3, 4, 5, 6, 7, 8, 9). In addition, histidine phosphorylation, like serine, threonine, and tyrosine phosphorylation, can regulate protein activity; well-studied examples of this type of posttranslational modification include proteins associated with bacterial phosphotransferase systems (2, 10) and the mammalian potassium channel KCa3.1 (11, 12, 13, 14, 15). Technical challenges have historically been a major obstacle in studying histidine phosphorylation (16, 17, 18), and recent studies suggest that this protein modification may be far more prevalent than has been previously appreciated (19, 20, 21, 22). In contrast to the very stable phosphoesters of serine, threonine, and tyrosine, which depend on phosphatases for dephosphorylation, the phosphoramidate of phosphohistidine can be quite labile (23, 24, 25, 26). For this reason, phosphohistidine phosphatases† may not be required for dephosphorylating phosphohistidine *in vivo* in many contexts. Nevertheless, phosphohistidine phosphatases have been identified. It has been known for some time that some phosphatases can dephosphorylate phosphohistidine *in vitro* (27, 28), but in most cases this activity has not yet been shown to be physiologically relevant. To our knowledge, there are only four phosphohistidine phosphatases for which *in vivo* targets have been proposed: SixA in *Escherichia coli* and PHPT1, PGAM5, and LHPP in mammals (21, 29, 30, 31, 32, 33, 34, 35, 36, 37).

SixA (**S**ignal **I**nhibitory factor **X**) is a well-conserved bacterial protein (38) belonging to the histidine phosphatase superfamily (1, 39, 40). The “histidine” in this superfamily name refers not to substrate specificity but rather to the conserved residue in the active site that is essential for enzyme activity and that becomes phosphorylated during catalysis. SixA was first reported to be a phosphohistidine phosphatase that dephosphorylates the histidine-containing phosphotransfer domain of the *E. coli* sensor kinase ArcB (32, 41), and the activity was shown to be dependent on the conserved active-site histidine (His8) of SixA. However, beyond the two reports that proposed this activity 2 decades ago, we are unaware of any other publications describing SixA regulation of ArcB. In fact, at least two subsequent studies failed to find an effect of SixA on ArcB-regulated transcription (33, 42). Recently, analysis of a growth defect of a SixA-null strain identified a different regulatory pathway that appears to be a physiological target of SixA (33): the nitrogen-related phosphotransferase system.

In contrast to the classical carbohydrate phosphotransferase systems, the nitrogen-related phosphotransferase system does not participate in sugar phosphorylation and uptake, but, like the carbohydrate systems, it regulates a diverse set of cellular processes, including potassium homeostasis, nitrogen and carbon metabolism, the stringent response, and two-component signaling (10, 43). In most cases, regulation is mediated by phosphorylation-dependent protein–protein interactions (2, 10, 43, 44, 45). In *E. coli*, the nitrogen-related phosphotransferase system consists of a phosphotransferase, EI^Ntr^, and two phosphocarriers, NPr and EIIA^Ntr^ (Fig. 1*A*). Together, these three proteins perform reversible phosphoryl transfer reactions *via* histidine residues in a similar fashion to their counterparts in carbohydrate phosphotransferase systems: a phosphoryl group is first transferred from phosphoenolpyruvate to EI^Ntr^, next from EI^Ntr^ to NPr, and finally from NPr to EIIA^Ntr^.

**Figure 1:**
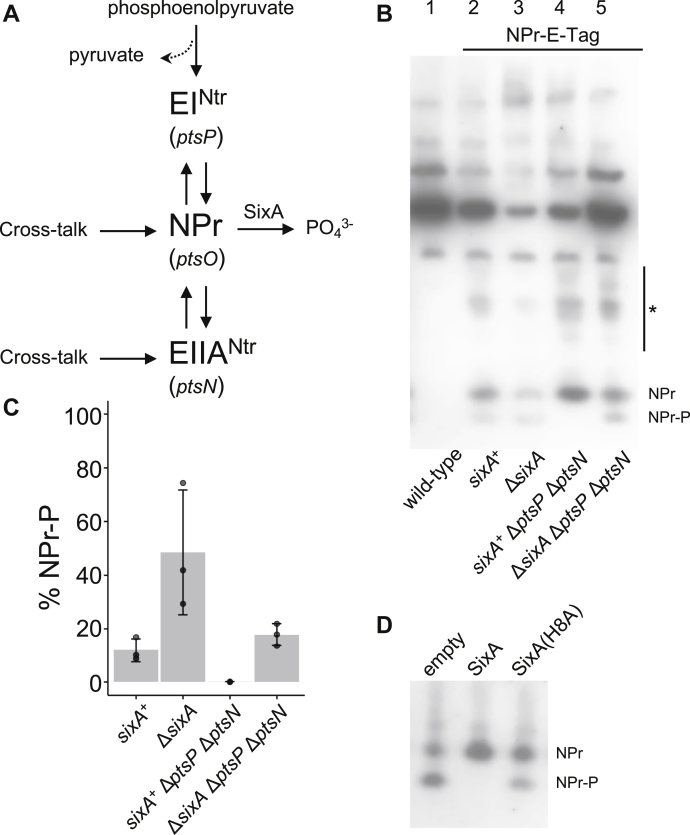
***E. coli* NPr is hyperphosphorylated when SixA is absent.***A*, schematic of phosphoryl transfer by the nitrogen-related phosphotransferase system, with a focus on sources of NPr phosphorylation and dephosphorylation. *Solid arrows* denote paths of phosphoryl transfer by EI^Ntr^, NPr, EIIA^Ntr^, and SixA, which all occur though phosphohistidine intermediates. The genes for each protein of the phosphotransferase system are indicated in parentheses. Cross talk refers to phosphorylation of NPr and EIIA^Ntr^ by other pathways. *B*, representative anti-E-Tag western blot of cell lysates separated by native gel electrophoresis. The asterisk indicates additional NPr-E-Tag-dependent bands (see text for discussion). Strains used in this experiment were MG1655, JES287, JES288, JES314, and JES315. C, band intensities of NPr and NPr-P from (B) and two additional blots were quantified with ImageJ (74); %NPr-P = 100 × NPr-P/(NPr + NPr-P). Columns of the bar graph indicate average %NPr-P for strains from the three independent experiments, error bars show one standard deviation, and symbols represent values from individual cultures. *D*, representative anti-E-Tag western blot of cell lysates from strains expressing NPr-E-Tag and either wild-type SixA or a catalytic mutant of SixA. Expression of SixA proteins was from leaky (*i.e.*, uninduced) transcription from the *trc* promoter on a plasmid. Strains used were JES288 (Δ*sixA* NPr-E-Tag^+^) transformed with empty vector pTrc99a, SixA-expressing plasmid pSixA, or SixA(H8A)-expressing plasmid pSixA(H8A).

Whereas carbohydrate phosphotransferase systems donate phosphoryl groups to incoming carbohydrates or acceptor proteins, the fate of the phosphoryl groups that pass through the nitrogen-related phosphotransferase system is not understood in most bacteria. Two exceptions are *Acinetobacter baumannii*, in which phosphoryl groups can be transferred from NPr to a serine residue of the protein GigB and subsequently removed by the phosphoserine phosphatase GigA (46), and *Sinorhizobium meliloti*, in which an aspartic acid residue near the phosphorylation site of NPr (called HPr in this organism) is proposed to heighten the autohydrolysis rate (47). While it is possible that autohydrolysis also plays a role in *E. coli*, recent work suggests that SixA provides another mechanism for dephosphorylating the nitrogen-related phosphotransferase system (33). In the absence of SixA, EIIA^Ntr^ becomes hyperphosphorylated. In addition, the reduction of EIIA^Ntr^ phosphorylation by SixA requires NPr. These observations, together with epistasis studies of a Δ*sixA* growth defect, led to the proposal that phosphorylated NPr (NPr-P) is a substrate for SixA.

Here we establish through *in vivo* and *in vitro* experiments that SixA is a phosphohistidine phosphatase for NPr-P. The NPr-P dephosphorylation reaction proceeds through an unstable phosphohistidine intermediate of SixA. We also show that the SixA–NPr interaction is important for *E. coli* to colonize its natural niche, the mammalian intestinal tract. These results establish a substrate for a highly conserved prokaryotic phosphohistidine phosphatase and identify a mechanism for modulating the activity of the nitrogen-related phosphotransferase system.

## Results

### SixA regulates NPr phosphorylation *in vivo*

To determine the effect of SixA on NPr phosphorylation, we analyzed strains expressing a tagged NPr (E-Tag) by western blot after native gel electrophoresis. Under nondenaturing conditions, the phosphorylated and nonphosphorylated forms of the protein are resolved into two bands, with the phosphorylated form corresponding to higher mobility (48), (Fig. 1*B*, lanes 2–5). Comparison of the results for the *sixA*^+^ and Δ*sixA* strains indicates that the fraction of NPr protein that is phosphorylated is increased when SixA is absent (Fig. 1*B*, lanes 2 and 3; Fig. 1*C*). We also note that there are several additional bands (with lower mobility) in the lanes with E-tagged NPr that are absent in the lane with untagged NPr (Fig. 1*B*, compare lanes 2–5 with lane 1). We do not know the identity of these additional bands, but they may indicate complexes of NPr with other proteins.

NPr is phosphorylated by EI^Ntr^ and also by reverse phosphotransfer from EIIA^Ntr^, which can be phosphorylated by cross talk from other phosphotransferase systems in mutant backgrounds (49) (Fig. 1*A*). Cross talk to NPr directly is also plausible (50) but, to our knowledge, has never been investigated *in vivo*. We therefore examined the effect of deleting *sixA* on NPr phosphorylation in strains that lack EI^Ntr^ and EIIA^Ntr^. A Δ*ptsP* Δ*ptsN* strain (EI^Ntr^-null, EIIA^Ntr^-null) had no detectable NPr-P (Fig. 1*B*, lane 4). In contrast, the triple-deletion Δ*sixA* Δ*ptsP* Δ*ptsN* showed considerable NPr phosphorylation (Fig. 1*B*, lane 5). These results establish that NPr can be phosphorylated by other pathways (possibly carbohydrate phosphotransferase systems) when EI^Ntr^, EIIA^Ntr^, and SixA are absent and further establish that SixA does not require EI^Ntr^ and EIIA^Ntr^ to modulate NPr phosphorylation.

We also tested whether SixA's active-site histidine is required for the protein to affect NPr phosphorylation by comparing the effects of expressing wild-type SixA or a SixA(H8A) mutant. Wild-type SixA expression eliminated NPr-P, whereas expression of SixA(H8A) did not (Fig. 1*D*). Collectively, the above results show that SixA modulates NPr phosphorylation independently of EI^Ntr^ and EIIA^Ntr^, and they support the hypothesis that SixA directly dephosphorylates NPr-P.

### SixA dephosphorylates NPr-P *in vitro* and forms a transient phosphohistidine intermediate

**Figure 2:**
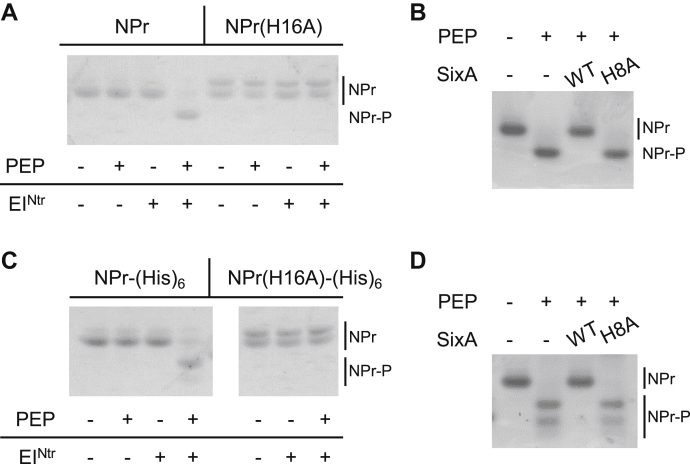
***in vitro* phosphorylation and dephosphorylation reactions of NPr assayed by native gel electrophoresis.***A*, Coomassie-stained native gel of NPr and NPr(H16A) phosphorylation reactions. Reactions contained 80 μM NPr or NPr(H16A) with or without 0.25 μM EI^Ntr^ and 5 mM phosphoenolpyruvate (PEP), as indicated, and were incubated for 30 min prior to electrophoresis. The two bands visible in the NPr(H16A) lanes do not appear to be due to two chemically distinct species—see text for discussion. *B*, Coomassie-stained native gel of NPr-P dephosphorylation reactions with wild-type SixA or a catalytic mutant SixA(H8A). NPr was phosphorylated as in (*A*) and then incubated with either SixA, SixA(H8A), or neither for 20 min prior to electrophoresis. Final concentrations of NPr and SixA/SixA(H8A) in the dephosphorylation reaction were 70 μM and 10 μM, respectively. *C*, Coomassie-stained native gel of NPr-(His)_6_ phosphorylation reactions. Phosphorylation reaction conditions for NPr-(His)_6_ and NPr(H16A)-(His)_6_ were as described in (*A*). Note that the image shows a single gel; the missing lanes are the lanes shown in (*A*). *D*, Coomassie-stained native gel of NPr-(His)_6_-P dephosphorylation reactions with wild-type SixA or a catalytic mutant SixA(H8A). The dephosphorylation reaction conditions were as described in (*B*). The NPr-(His)_6_-P doublets in (*C*) and (*D*) are discussed in the text.

To study the activity of SixA against NPr-P *in vitro*, we used purified NPr that consisted of the complete amino acid sequence of the protein plus eight additional residues at the C-terminus (see Experimental procedures). We will refer to this protein as “wild-type” NPr. We phosphorylated NPr by incubating with EI^Ntr^ and phosphoenolpyruvate. As expected (51, 52), phosphorylation required His16 of NPr (Fig. 2*A*, compare lanes 4 and 8). Curiously, the H16A mutant ran as a doublet on native gels; we will discuss this observation further below. Addition of purified SixA to NPr-P resulted in complete dephosphorylation of the protein, whereas the catalytic mutant SixA(H8A) had no observable effect on NPr-P level (Fig. 2*B*).

For the experiments described above and below, the polyhistidine tag used to purify NPr was cleaved from the protein. We had noticed that phosphorylation reactions of NPr-(His)_6_, a protein consisting of the complete amino acid sequence of NPr plus the eight additional residues LEHHHHHH, produced multiple bands (Fig. 2*C* lane 4; Fig. 2*D* lanes 2 and 4). In addition, incubation with SixA eliminated these bands, leaving only the band corresponding to unphosphorylated NPr-(His)_6_ (Fig. 2*D*). To determine whether these multiple bands correspond to multiple phosphorylation states of the protein, we analyzed NPr-(His)_6_ phosphorylation and mock-phosphorylation samples by mass spectrometry. Monoisotopic masses of phosphorylated NPr-(His)_6_ measured by electrospray ionization MS matched the masses for singly and doubly phosphorylated protein (Fig. S1).

Unfortunately, LC-MS/MS produced spectra of insufficient quality to assign phosphorylation to specific residues (data not shown), despite efforts to preserve acid-labile histidine phosphorylation. Since the extra bands appear only for the construct with the polyhistidine tag, and since these bands were eliminated after incubation with SixA (Fig. 2*D*), we suspect that one or more histidines in the (His)_6_ tag were phosphorylated. We further suspect that the (His)_6_ tag is phosphorylated by intramolecular phosphoryl transfer, because, first, the site of NPr phosphorylation, His16, is adjacent to the C-terminus of NPr (53, 54), and, second, NPr(H16A)-(His)_6_ shows no evidence of phosphorylation (Fig. 2*C*, lane 7). We are unaware of previous reports of histidine phosphorylation of polyhistidine tags, but we speculate that this reaction may occur if the polyhistidine tag is near a site of histidine phosphorylation of the native protein.

As noted above, NPr(H16A) protein preparations ran as doublets on native gels (Fig. 2*A*, lanes 5–8; Fig. 2*C*, lanes 5–7). The two bands are unlikely to reflect two chemically distinct forms of the protein because NPr(H16A) runs as a single band on SDS-PAGE (Fig. S2) and analysis of NPr(H16A) by electrospray ionization MS identified only a single species (data not shown). Although wild-type NPr protein preparations primarily ran as a single band with mobility similar to the higher mobility band of NPr(H16A), a faint lower mobility band for the wild-type protein was also discernable (for example Fig. 2*A*, lanes 1–3; Fig. 2*C*, lanes 1–4). The doublet on native gels may therefore indicate that our recombinant NPr has two stable (or quasi-stable) conformations. Indeed, previous work has also reported evidence for two conformations of recombinant NPr (54). Based on NMR spectroscopy, it was suggested that the two protein forms could be due to interactions with a disordered C-terminal tail containing additional residues not found in native NPr. Thus, it is possible that the doublet we observe on native gels is also due to the presence of additional residues at the C-terminus of the NPr proteins described above.

**Figure 3:**
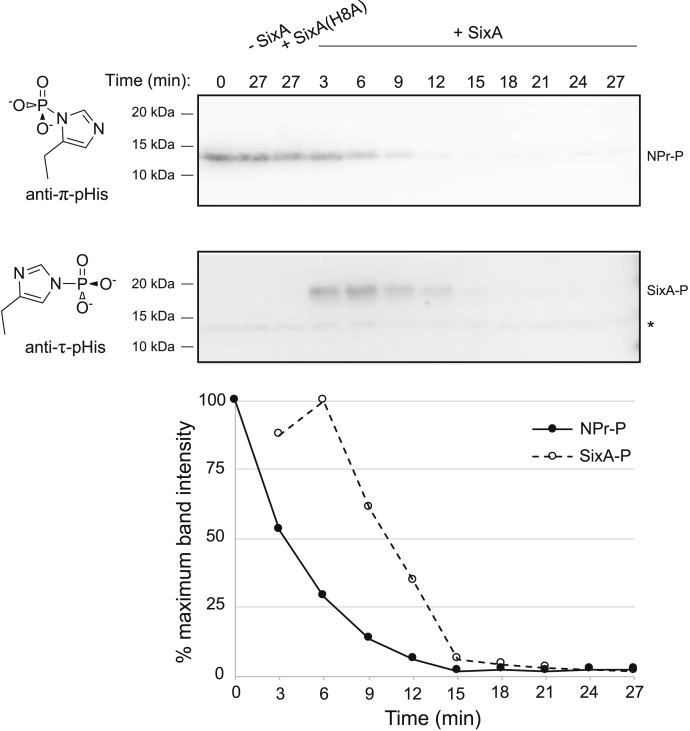
**Time course of NPr-P dephosphorylation by SixA assayed by SDS-PAGE and anti-phosphohistidine western blot.** Representative western blots of NPr-P dephosphorylation time course. NPr was phosphorylated in a reaction containing 20 μM NPr, 0.25 μM EI^Ntr^, and 5 mM phosphoenolpyruvate, and then NPr-P was separated from EI^Ntr^ by ultrafiltration and purified from phosphoenolpyruvate as described in Experimental procedures. SixA or SixA(H8A) was added to purified NPr-P at a concentration of 150 nM. *lane 1*—0 min time point taken prior to SixA addition; *lane 2*—27 min after addition of buffer without SixA; *lane 3*—27 min after addition of SixA(H8A); *lanes 4 to 12*—samples taken at indicated time points after SixA addition. The graph presents quantification of band intensities from the blots for the +SixA samples. The two control samples—addition of buffer without SixA and addition of SixA(H8A)—are not shown in the graph but had 86% and 64% maximum band intensity, respectively. Approximate molecular weight positions are indicated to the left of the blots. The asterisk by the anti-τ-pHis blot marks the location of a faint band discussed further in the text.

To follow the time course of the NPr-P dephosphorylation reaction, we turned to denaturing gels so that reactions could be quenched with denaturing loading dye. To detect phosphorylated NPr, we used antibodies against phosphohistidine. Histidine can be phosphorylated on either of the two nitrogen atoms of its imidazole ring, forming phosphoramidates called π-phosphohistidine (π-pHis) and τ-phosphohistidine (τ-pHis) (Fig. 3) (23, 24, 26). Based on western blots with phosphohistidine-isomer-specific antibodies, NPr-P consists primarily of π-pHis (Fig. 3). Following addition of SixA, NPr-P showed progressive loss of the π-pHis band (Fig. 3, 3 min–27 min). This decrease in π-pHis required addition of SixA and was not observed for mutant SixA(H8A) (Fig. 3, lanes 2 and 3). In addition, a band at the expected location for SixA appeared on the anti-τ-pHis blot at the time points corresponding to NPr-P dephosphorylation, and this band disappeared as NPr-P was dephosphorylated (Fig. 3, 3 min–27 min). These results indicate that NPr-P dephosphorylation by SixA proceeds *via* a transient SixA phosphohistidine intermediate.

The anti-τ-pHis blot also showed a faint band at the expected location of NPr (Fig. 3, asterisk). In addition, the anti-π-pHis blot shows very faint bands at the position for NPr-P even at the latest time points in the blot. These weak bands were still detectable when samples were boiled to eliminate phosphohistidine (Fig. S3), suggesting that they may reflect weak antibody binding to unphosphorylated NPr rather than true phosphohistidine signal. Interestingly, we do not detect a comparable signal when the sole histidine residue of NPr is mutated to alanine (Fig. S3), which could indicate weak cross-reactivity of the antibodies with unphosphorylated histidine on NPr.

From the *in vitro* results presented above, we conclude that SixA directly dephosphorylates NPr-P, and the reaction proceeds by phosphoryl transfer from π-pHis on NPr to τ-pHis on SixA.

### A *sixA* deletion has an NPr-dependent colonization defect in the mouse intestine

**Figure 4:**
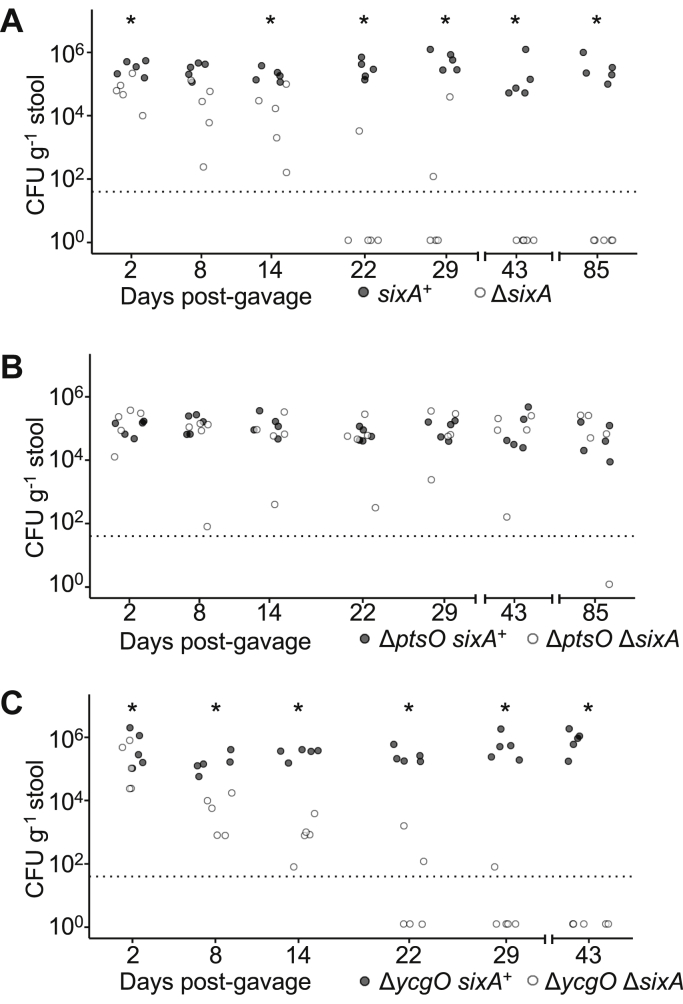
**Mouse colonization competitions of Δ*sixA E. coli* reveal an NPr-dependent defect.***A*, competition between wild-type and Δ*sixA* strains derived from *E. coli* MP1 (MP7 and MP204). *B*, competition between Δ*ptsO* (*ptsO* encodes NPr) and Δ*ptsO* Δ*sixA* strains (MP274 and MP271). *C*, competition between Δ*ycgO* and Δ*ycgO* Δ*sixA* strains (MP275 and MP273). All graphs: symbols indicate the fecal load of *E. coli* for individual mice, and dashed lines indicate the detection limit of 40 colony forming units (CFU) g^−1^ stool. Asterisks indicate *p* < 0.05 significance, as assessed by the Wilcoxon test for paired samples, after normalizing fecal load measurements by relative amounts of the two strains in the solution used to gavage mice.

Since animal intestinal tracts are one of the primary niches for *E. coli*, we wondered whether SixA is important for *E. coli* fitness in this environment. We took advantage of a previously developed system for studying *E. coli* intestinal colonization (55) that uses a mouse *E. coli* commensal isolate, MP1. This strain is genetically tractable and does not require continuous antibiotic treatment for long-term persistence in the mouse intestine. We found that the Δ*sixA* strain had a colonization defect in competition with wild-type (Fig. 4*A*). Six weeks after orogastric gavage of a suspension of wild-type and mutant bacteria, counts of the Δ*sixA* strain were below the detection limit of our assay for all mice. In contrast, the wild-type strain stably colonized mice for 12 weeks.

Based on the results above, we hypothesized that the Δ*sixA* colonization defect is due to hyperphosphorylation of NPr (encoded by *ptsO*). We therefore tested whether deletion of *sixA* also affects colonization of an NPr-null strain. In competition experiments, counts of Δ*ptsO* and Δ*ptsO* Δ*sixA* strains were comparable for four of the five mice 12 weeks after gavage (Fig. 4*B*). These results are consistent with our conclusion above that SixA dephosphorylates NPr-P and also with the hypothesis that the colonization defect is due to aberrant NPr-P dephosphorylation.

Previously, we showed that the absence of SixA causes a growth defect in minimal medium but not in rich medium (33) (see also Fig. S4). The slow-growth phenotype is suppressed by deleting *ycgO*, which encodes a putative cation–proton antiporter that has been proposed to be regulated by the nitrogen-related phosphotransferase system (56). To test whether the Δ*sixA* mouse colonization defect also involves YcgO, we performed competitions between Δ*ycgO* and Δ*ycgO* Δ*sixA* strains. Six weeks after gavage, counts of the Δ*ycgO* Δ*sixA* strain were below the detection limit of our assay, whereas the Δ*ycgO* strain continued to stably colonize mice (Fig. 4*C*). From these results we conclude that the Δ*sixA* mouse colonization defect is not related to the YcgO-dependent slow-growth phenotype of Δ*sixA E. coli* in minimal medium. Taken together, our results suggest that the colonization defect arises from the absence of SixA dephosphorylation of NPr-P. The resulting hyperphosphorylation of one or more members of the nitrogen-related phosphotransferase system then leads to misregulation of a protein target other than YcgO.

## Discussion

Here we have demonstrated that SixA dephosphorylates the phosphocarrier NPr, establishing a phosphatase-based mechanism for controlling the phosphorylation state of the nitrogen-related phosphotransferase system. Across different bacteria, this phosphotransferase system has been shown to regulate diverse metabolic pathways and to affect host colonization by pathogens and symbionts (10, 43, 57, 58). Our mouse experiments, which revealed that eliminating SixA impaired *E. coli* colonization though an NPr-dependent mechanism, provide another example of a role for the nitrogen-related phosphotransferase system in host–microbe interactions. We note that this Δ*sixA* mouse colonization defect is characterized by a gradual decrease in mutant bacteria over time, and we cannot rule out the possibility that this phenotype is due to a subtle growth defect that also exists outside of an animal host. However, this colonization defect is distinct from a previously described growth defect of the Δ*sixA* mutant *in vitro*. This latter defect, which we observe in minimal medium but not in rich medium, is suppressed by deleting *ycgO* (33), a gene that is predicted to encode a proton–cation antiporter. The mouse colonization defect, on the other hand, is not affected by Δ*ycgO* (Fig. 4*C*).

**Figure 5:**
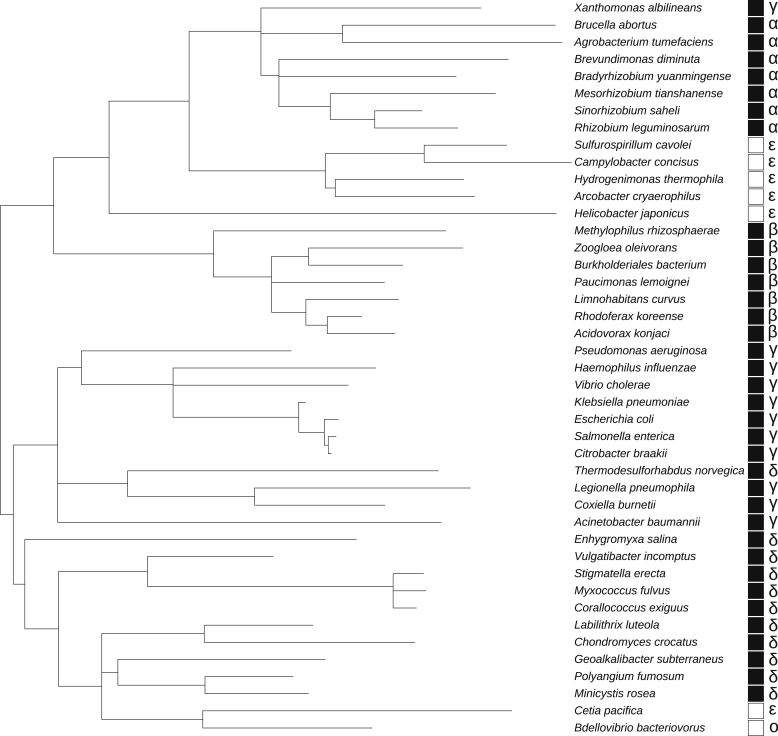
**Patterns of co-occurrence between SixA and NPr homologs in Proteobacteria species.** SixA protein sequences from a selection of Proteobacteria were used to construct a tree as described in Experimental procedures. The *black and white squares* on the right indicate whether a species has proteins with homology to NPr/HPr—presence (*black*) or absence (*white*) of NPr/HPr-like proteins. The Proteobacteria class of each species is indicated at the far right by the Greek letters (alpha, beta, gamma, delta, epsilon) or, for Oligoflexia, the letter “O.”

SixA homologs are distributed across many phyla and are especially well represented among Proteobacteria, Cyanobacteria, and Actinobacteria (38). Within Proteobacteria, SixA sequences tend to cluster together by taxonomic class (Fig. 5). In addition, some bacterial genomes that encode SixA homologs appear to lack genes encoding phosphocarrier proteins (NPr, HPr, or proteins containing an NPr/HPr-like domain), *e.g.*, *Bdellovibrio bacteriovorus* and members of Epsilonproteobacteria (Fig. 5) as well as Cyanobacteria and *Mycobacterium* species. Therefore, at least some SixA homologs, if they function as phosphatases, must target substrates other than phosphocarrier proteins. Of course, it is also possible that *E. coli* SixA has other targets in addition to NPr.

Using antibodies specific to either τ-pHis or π-pHis isomers, our western blot analyses indicate that SixA dephosphorylates π-pHis of NPr (Fig. 3). Studies of NPr homologs have previously observed that these proteins are phosphorylated at the π-position (59, 60). Our results also revealed that during the dephosphorylation reaction, SixA reacts with τ-pHis antibody (Fig. 3). This finding is in agreement with predictions that catalysis by SixA proceeds through a τ-pHis intermediate (38, 39). Together, these two observations indicate that phosphoryl transfer from NPr to SixA involves switching from π-pHis (on NPr) to τ-pHis (on SixA). Interestingly, several other phosphoryl transfer reactions between histidine residues also involve switching between phosphohistidine isomers (τ-pHis → π-pHis, or π-pHis → τ-pHis). For example, phosphoryl transfer through the bacterial carbohydrate phosphotransferase system proceeds *via* the sequence: τ-pHis (EI) → π-pHis (HPr) → τ-pHis (EIIA) (see (45) for references and additional phosphotransferase system examples). In addition, in mammalian cells, π-pHis → τ-pHis phosphoryl transfer is observed when the phosphohistidine phosphatase PGAM5 dephosphorylates NDPK-B (31) and also when NDPK-B phosphorylates the potassium channel KCa3.1 (12). More examples can be found in (61, 62). From our survey of the phosphohistidine literature, we were unable to find examples of π-pHis → π-pHis or τ-pHis → τ-pHis phosphoryl transfer. It is an interesting question whether the above pattern of switching between the π-pHis and τ-pHis isomers arises from a general mechanistic constraint on pHis → pHis phosphoryl transfer or instead reflects some aspect of the evolution of the associated pathways.

Although SixA was discovered 2 decades ago, phosphohistidine phosphatases remain relatively understudied. In addition to the target for SixA described here, physiological targets of the mammalian phosphohistidine phosphatases PHPT1, PGAM5, and LHPP have only recently been uncovered (21, 29, 30, 31). Further study of these four proteins, as well as the discovery of additional phosphohistidine phosphatase substrates, will reveal new biological processes that exploit histidine phosphorylation in both prokaryotes and eukaryotes.

## Experimental procedures

Strains, plasmids, and primers are listed in Tables S1–S3 (63, 64, 65), respectively.

### Growth conditions

Bacterial cultures were grown at 37 °C with aeration in Lysogeny Broth (LB) Miller medium (66), which contains (per liter) 10 g tryptone, 5 g yeast extract, and 10 g NaCl (catalog no. BP1426-500; Fisher Scientific). Antibiotics were used at the following concentrations (in μg/ml): ampicillin, 100; kanamycin, 25 or 50; chloramphenicol, 12; and tetracycline, 15. Strains expressing proteins from the *trc* promoter (P_*trc*_) were grown in the absence of inducer (IPTG; isopropyl-β-D-thiogalactopyranoside); expression was based on leaky transcription from P_*trc*_.

### Strain construction

Antibiotic-resistance cassettes flanked by FLP recombination target (FRT) sites were excised from bacterial genomes using FLP recombinase expressed from pCP20 (67). Phage transduction was performed using P1_vir_ (66). Recombineering was performed following previously published protocols (68) with PCR fragments and strains transformed with pKD46, as described in more detail below.

JES286 is a strain with a C-terminal epitope sequence (E-Tag) fused to the NPr gene *ptsO* at its chromosomal locus in *E. coli* MG1655. A PCR fragment, containing the end of *ptsO* fused to a sequence encoding the E-Tag (GAPVPYPDPLEPR) and also followed by a FLP-recombinase-excisable *cat* gene, was constructed by PCR with the template pKD3 and with primers npr-Etag-lred-F/R. The resulting DNA segment was electroporated into strain MG1655/pKD46, and cells were selected on chloramphenicol. The *ptsO* locus of JES286 was confirmed by DNA sequencing. To ensure selection of both the *ptsN* deletion and the *ptsO* E-Tag fusion during construction of JES314 and JES315, transductants were selected on agar plates containing both chloramphenicol and kanamycin.

MP1 deletion strains were constructed by electroporating MP1/pKD46 with PCR products and selecting for mutants on kanamycin. Electrocompetent MP1/pKD46 was prepared as previously described (55), except cells were washed with an ice cold solution containing 10% glycerol and 1 mM unbuffered 3-(N-morpholino) propanesulfonic acid (MOPS). To construct the *sixA*-deletion strain, MP203, a PCR fragment containing FRT-*kan*-FRT was amplified from strain JW2337 with primers sixA-del-U1/L1. To construct the *ptsO*-deletion strain, MP263, a PCR fragment containing FRT-*kan*-FRT was amplified from pKD13 with primers MP1-npr-lred-u/d2. Finally, to construct the *ycgO*-deletion strain, MP264, a PCR fragment containing FRT-*kan*-FRT was amplified from pKD13 with primers MP1-ycgO-lred-u/d2. Deletions in MP1 were confirmed by PCR and moved into the MP1 derivatives by P1 transduction as indicated in Table S1. Out of convenience for strain construction, the *ptsO*- and *ycgO*-deletion strains used in mouse competitions were not constructed in the order that would ensure the closest possible common ancestor between the *sixA*^+^ and *sixA*^-^ derivatives (Table S1).

### Assays of NPr phosphorylation *in vivo*

Cell pellets from 2 ml of stationary-phase cultures were used to prepare cleared cell lysates by either (1) resuspending pellets in 0.5 ml of loading dye (10% glycerol, 40 mM glycine, 5 mM Tris HCl, 0.005% bromophenol blue, pH 8.8), sonicating suspensions on ice, and centrifuging cell lysates to pellet cell debris or (2) resuspending pellets in 0.25 ml B-PER (Bacterial Protein Extraction Reagent; catalog no. 78248; Thermo Fisher Scientific), incubating at room temperature for 15 min, and mixing cleared lysate with an equal volume of loading dye. Samples were then analyzed by native gel electrophoresis on 4% to 20% Mini-PROTEAN TGX precast protein gels (catalog no. 4561096; Bio-Rad Laboratories) with running buffer consisting of 25 mM Tris, 192 mM glycine, pH 8.3. Electrophoresis was performed at room temperature or 4 °C until the loading dye reached the end of the gel. Protein transfer to 0.45-μm-pore-size Immobilon-P PVDF (polyvinylidene difluoride) membrane (catalog no. IPVH00010; Millipore) was performed with transfer buffer containing 20% methanol, 25 mM Tris, 192 mM glycine, pH 8.3.

Membranes were blocked with 5% milk TBST (Tris-buffered saline and Tween solution; contains [per liter] 8 g NaCl, 0.38 g KCl, 3 g Tris base, 500 μl Tween 20, pH 7.4). Rabbit anti-E-Tag (catalog no. A190-133A, RRID: AB_345221; Bethyl Laboratories) was diluted in blocking buffer to 0.3 to 1.0 μg/ml. Anti-rabbit-horseradish peroxidase (catalog no. R1006; Kindle Biosciences) was diluted 1:1000 in blocking buffer. Enhanced chemiluminescence (ECL) detection was performed with KwikQuant Ultra Digital-ECL Substrate (catalog no. R1004; Kindle Biosciences), and membranes were imaged with a KwikQuant Imager (Kindle Biosciences).

### Expression plasmid construction

To construct the NPr-(His)_6_ expression plasmid pJS43, primers NdeI-ptsO-F and XhoI-ptsO-R were used to amplify the *ptsO* gene from MG1655 genomic DNA. The resulting DNA segment was digested with NdeI and XhoI and cloned into pET-22b(+), which was digested with the same enzymes. A plasmid expressing the H16A *ptsO* mutant was constructed by site-directed mutagenesis: plasmid pJS43 was amplified with primers npr-H16A-F/R, the ends were phosphorylated with T4 polynucleotide kinase, and the resulting DNA was circularized by blunt-end ligation with T4 DNA ligase, generating pJS47. NPr-(His)_6_ and NPr(H16A)-(His)_6_ expressed from these plasmids consist of the complete NPr sequence plus the additional residues LEHHHHHH at the C-terminus.

To purify NPr and SixA proteins with cleavable polyhistidine tags, genes were cloned into the pET-41 vector backbone using NEBuilder HiFi DNA Assembly (New England BioLabs). Each fusion protein contains the complete protein sequence plus the additional residues GTENLYFQGSTMDHHHHHHHH at the C-terminus. After removing the polyhistidine tag with TEV (tobacco etch virus) protease, the residues GTENLYFQ remain at the C-terminus.

The NPr-TEV-(His)_8_ expression plasmid pJS65 was constructed with a two-piece assembly; the vector backbone was amplified from pEKS001 using primers ptsO-TEV-vec/ptsO-pEKS-vec, and the insert was amplified from MG1655 genomic DNA using primers ptsO-pEKS-ins/ptsO-TEV-ins. The NPr(H16A)-TEV-(His)_8_ expression plasmid pJS69 was constructed with a three-piece assembly. pJS65 was used as the template for all three PCR reactions: the 5’ end of *ptsO* was amplified with primers pBR322-seq-Rev/NPr-H16A-R1, the 3’ end of *ptsO* was amplified with primers NPr-H16A-F2/pET-seq-F, and the vector backbone was amplified with primers pBR322-seq-Rev-comp/pET-seq-F-comp.

The SixA-TEV-(His)_8_ expression plasmid pJS67 was constructed with a two-piece assembly. The vector backbone was amplified from pEKS001 using primers sixA-TEV-vec/sixA-pEKS-vec, and the insert was amplified from MG1655 genomic DNA using primers sixA-pEKS-ins/sixA-TEV-ins. The SixA(H8A)-TEV-(His)_8_ expression plasmid pJS70 was constructed with a three-piece assembly; the vector backbone was amplified from pJS65 using primers pBR322-seq-Rev-comp/pET-seq-F-comp, the 5’ end of *sixA* was amplified from pJS67 with primers pBR322-seq-Rev/SixA-H8A-R1, and the 3’ end of *sixA* was amplified from pJS67 with primers SixA-H8A-F2/pET-seq-F.

The insertions in all engineered plasmids were verified to be correct by DNA sequencing.

### Protein purification

Proteins were expressed in BL21(DE3) and purified using Ni-NTA agarose (catalog no. 30210; QIAGEN) following the manufacturer’s protocols. Protein expression was induced with 1 mM IPTG at mid- to late-exponential phase and grown for an additional 3 to 4 h at 37 °C. Cells were lysed by sonication on ice or by suspension in B-PER. Cleavable histidine tags were removed by incubation with His-tagged TEV protease (catalog no. T4455; Sigma), and tag-free protein was purified from the reaction by incubating with Ni-NTA agarose and recovering the flow-through. Proteins were transferred into storage buffer (50 mM Tris HCl, 150 mM NaCl, 1 mM DTT, 10% glycerol, pH 8) by either dialysis or with centrifugal filter units (3 kDa cutoff; Amicon UFC800308, Millipore-Sigma). The storage buffer for NPr proteins also contained 5 mM MgCl_2_. Protein concentrations were estimated with the Bradford method (Bio-Rad Protein Assay 500-0006, Bio-Rad Laboratories) using a standard curve with BSA. Proteins were stored at −80 °C.

### NPr phosphorylation assay

To prepare phosphorylated NPr, phosphoenolpyruvate, NPr, and EI^Ntr^ (a gift from Alan Peterkofsky) were first incubated individually in reaction buffer (50 mM Tris HCl, 5 mM MgCl_2_, 2 mM DTT, pH 8) at 30 °C for 15 min (46). After preincubation, reaction components were then mixed together and incubated for 30 min at 30 °C. Protein and phosphoenolpyruvate concentrations in phosphorylation reactions are indicated in the figure captions.

Native gel electrophoresis to analyze NPr phosphorylation was performed as described above for cleared cell culture lysates, except samples were mixed with 2× nondenaturing, reducing loading dye (20% glycerol, 80 mM glycine, 10 mM Tris HCl, 100 mM DTT, 0.01% bromophenol blue, pH 8.8), and gels were stained for protein with Coomassie Brilliant Blue R-250 (#161-0400, Bio-Rad Laboratories).

### NPr-P dephosphorylation assay

For the dephosphorylation reactions shown in Figure 2, aliquots of NPr phosphorylation reactions were mixed with SixA, SixA(H8A), or buffer alone and incubated at 30 °C for an additional 20 min before analysis by native gel electrophoresis.

To follow NPr-P dephosphoryation over time in Figure 3, NPr-P was purified by processing the phosphorylation reaction through a centrifugal filter unit (30 kDa cutoff; Amicon UFC503008, Millipore-Sigma) to eliminate EI^Ntr^, concentrating the flow-through 2.5-fold with a centrifugal filter unit (3 kDa cutoff; Amicon UFC503008, Millipore-Sigma), and then spinning the ultrafiltrate through a desalting spin column (Zeba 89882, Thermo Scientific) to reduce phosphoenolpyruvate levels. Dephosphorylation reactions contained 150 nM SixA, SixA(H8A), or neither and were incubated at 30 °C. Samples at each time point were quenched by mixing with 3× SDS-PAGE loading dye (6% SDS, 30% glycerol, 195 mM Tris HCl, 15% β-mercaptoethanol, 0.015% bromophenol blue, pH 8.8).

Quenched samples were separated by electrophoresis on duplicate gels as described above, except that denaturing conditions were used with SDS-PAGE running buffer (0.1% SDS, 25 mM Tris, 192 mM glycine, pH 8.3). After protein transfer, the two membranes were blocked in 3% milk TBST. Each membrane was probed with only one of the two anti-phosphohistidine antibodies (catalog no. ZRB1330, RRID: AB_2868462; catalog no. ZRB1352, RRID: AB_2868463; Millipore-Sigma), which were diluted in TBS (TBST lacking Tween) to 0.6 μg/ml. ECL detection was performed as described above.

### Mouse colonization

All animal studies were performed in accordance with animal protocols approved by the Institutional Animal Care and Use Committee of the University of Pennsylvania. Ten-week-old female C57BL/6 mice (The Jackson Laboratory) were raised under standard conditions. Bacterial suspensions for mouse inoculation were prepared as described previously (55). The suspension, containing a 1:1 mixture of two bacterial strains, was used to inoculate five cohoused mice by orogastric gavage with approximately 10^9^ bacteria. The ratio of Δ*sixA* to *sixA*^+^ bacteria in the solution used to gavage mice ranged between 0.7 and 1.1. Colony forming units (CFUs) were determined by stool serial dilution plating on selective agar media, as previously described (55). In brief, stool dilutions were plated on LB agar plates containing tetracycline to induce fluorescent protein production. Colonies were imaged with a home-built fluorescence imaging system, and the competing strains were distinguished by mCherry or GFP fluorescence. When Δ*sixA* strains were less abundant at later time points, stool suspensions were also plated on LB agar plates containing both tetracycline and kanamycin to count the GFP^+^ kanamycin-resistant colonies of Δ*sixA* strains.

### SixA phylogenetic tree

SixA phylogenetic tree MEGA-X (69) and NCBI BLAST (70) were used to prepare a maximum likelihood tree of SixA sequences as described in (71). The tree was visualized and annotated using iTOL (72). SixA homologs were identified based on the amino acid sequence determinants described in (38). NPr/HPr homologs or NPr/HPr-like domains in fusion proteins were identified based on the well-conserved signature sequence surrounding the active-site histidine residue (73). Table S4 contains the strains, NCBI taxonomic identifier, and protein accession information for SixA homologs and NPr/HPr homologs, if present. In the cases where more than one SixA homolog or NPr-like protein is present in a bacterial strain, only one representative sequence was taken.

## Data availability

Requests for data are to be addressed to the corresponding author (Mark Goulian, goulian@sas.upenn.edu).

## Conflicts of interest

The authors declare that they have no conflicts of interest with the contents of this article.

## Footnotes

† Strictly speaking, the name phosphoramidatase should be used, because it is a P–N bond, rather than a P–O bond, that is hydrolyzed (28). We will nonetheless adhere to the more commonly used term “phosphohistidine phosphatase.”

## References

[1] Rigden D.J. (2008). The histidine phosphatase superfamily: structure and function. Biochem. J..

[2] Deutscher J., Francke C., Postma P.W. (2006). How phosphotransferase system-related protein phosphorylation regulates carbohydrate metabolism in bacteria. Microbiol. Mol. Biol. Rev..

[3] Attwood P.V., Muimo R. (2018). The actions of NME1/NDPK-A and NME2/NDPK-B as protein kinases. Lab. Invest..

[4] Stock A.M., Robinson V.L., Goudreau P.N. (2000). Two-component signal transduction. Annu. Rev. Biochem..

[5] Bridger W.A., Boyer P.D. (1974). Succinyl-coA synthetase. Protein Synthesis, DNA Synthesis and Repair, RNA Synthesis, Energy-Linked ATPase Synthetases.

[6] Williams S.P., Sykes B.D., Bridger W.A. (1985). Phosphorus-31 nuclear magnetic resonance study of the active site phosphohistidine and regulatory phosphoserine residues of rat liver ATP-citrate lyase. Biochemistry.

[7] Carroll L.J., Xu Y., Thrall S.H., Martin B.M., Dunaway-Mariano D. (1994). Substrate binding domains in pyruvate phosphate dikinase. Biochemistry.

[8] Bridger W.A., Societies P.-A. A. o. B. (1973). Participation of enzyme-bound phosphohistidine in phosphate cleavage and transfer reactions. PAABS Revista.

[9] Schneider F. (1978). Histidine in enzyme active centers. Angew. Chem. Int. Ed. Engl..

[10] Galinier A., Deutscher J. (2017). Sophisticated regulation of transcriptional factors by the bacterial phosphoenolpyruvate: sugar phosphotransferase system. J. Mol. Biol..

[11] Srivastava S., Li Z., Ko K., Choudhury P., Albaqumi M., Johnson A.K., Yan Y., Backer J.M., Unutmaz D., Coetzee W.A., Skolnik E.Y. (2006). Histidine phosphorylation of the potassium channel KCa3.1 by nucleoside diphosphate kinase B is required for activation of KCa3.1 and CD4 T cells. Mol. Cell.

[12] Srivastava S., Panda S., Li Z., Fuhs S.R., Hunter T., Thiele D.J., Hubbard S.R., Skolnik E.Y. (2016). Histidine phosphorylation relieves copper inhibition in the mammalian potassium channel KCa3.1. Elife.

[13] Buljubasic F., El-Battrawy I., Lan H., Lomada S.K., Chatterjee A., Zhao Z., Li X., Zhong R., Xu Q., Huang M., Liao Z., Lang S., Cyganek L., Zhou X., Wieland T. (2020). Nucleoside diphosphate kinase B contributes to arrhythmogenesis in human-induced pluripotent stem cell-derived cardiomyocytes from a patient with arrhythmogenic right ventricular cardiomyopathy. J. Clin. Med..

[14] Di L., Srivastava S., Zhdanova O., Sun Y., Li Z., Skolnik E.Y. (2010). Nucleoside diphosphate kinase B knock-out mice have impaired activation of the K^+^ channel KCa3.1, resulting in defective T cell activation. J. Biol. Chem..

[15] Zhou X.B., Feng Y.X., Sun Q., Lukowski R., Qiu Y., Spiger K., Li Z., Ruth P., Korth M., Skolnik E.Y., Borggrefe M., Dobrev D., Wieland T. (2015). Nucleoside diphosphate kinase B-activated intermediate conductance potassium channels are critical for neointima formation in mouse carotid arteries. Arterioscler. Thromb. Vasc. Biol..

[16] Fuhs S.R., Hunter T. (2017). pHisphorylation: the emergence of histidine phosphorylation as a reversible regulatory modification. Curr. Opin. Cell Biol..

[17] Matthews H.R. (1995). Protein kinases and phosphatases that act on histidine, lysine, or arginine residues in eukaryotic proteins: a possible regulator of the mitogen-activated protein kinase cascade. Pharmacol. Ther..

[18] Fujitaki J.M., Smith R.A. (1984). Techniques in the detection and characterization of phosphoramidate-containing proteins. Methods Enzymol..

[19] Hardman G., Perkins S., Brownridge P.J., Clarke C.J., Byrne D.P., Campbell A.E., Kalyuzhnyy A., Myall A., Eyers P.A., Jones A.R., Eyers C.E. (2019). Strong anion exchange-mediated phosphoproteomics reveals extensive human non-canonical phosphorylation. EMBO J..

[20] Potel C.M., Lin M.H., Heck A.J.R., Lemeer S. (2018). Widespread bacterial protein histidine phosphorylation revealed by mass spectrometry-based proteomics. Nat. Methods.

[21] Hindupur S.K., Colombi M., Fuhs S.R., Matter M.S., Guri Y., Adam K., Cornu M., Piscuoglio S., Ng C.K.Y., Betz C., Liko D., Quagliata L., Moes S., Jenoe P., Terracciano L.M. (2018). The protein histidine phosphatase LHPP is a tumour suppressor. Nature.

[22] Fuhs S.R., Meisenhelder J., Aslanian A., Ma L., Zagorska A., Stankova M., Binnie A., Al-Obeidi F., Mauger J., Lemke G., Yates J.R., Hunter T. (2015). Monoclonal 1- and 3-phosphohistidine antibodies: new tools to study histidine phosphorylation. Cell.

[23] Attwood P.V., Piggott M.J., Zu X.L., Besant P.G. (2007). Focus on phosphohistidine. Amino Acids.

[24] Kee J.M., Muir T.W. (2012). Chasing phosphohistidine, an elusive sibling in the phosphoamino acid family. ACS Chem. Biol..

[25] Hultquist D.E., Moyer R.W., Boyer P.D. (1966). The preparation and characterization of 1-phosphohistidine and 3-phosphohistidine. Biochemistry.

[26] Makwana M.V., Muimo R., Jackson R.F. (2018). Advances in development of new tools for the study of phosphohistidine. Lab. Invest..

[27] Attwood P.V. (2013). P-N bond protein phosphatases. Biochim. Biophys. Acta.

[28] Jung H., Shin S.H., Kee J.M. (2019). Recent updates on protein *N*-phosphoramidate hydrolases. ChemBioChem.

[29] Srivastava S., Li Z., Soomro I., Sun Y., Wang J., Bao L., Coetzee W.A., Stanley C.A., Li C., Skolnik E.Y. (2018). Regulation of K_ATP_ channel trafficking in pancreatic β-cells by protein histidine phosphorylation. Diabetes.

[30] Srivastava S., Zhdanova O., Di L., Li Z., Albaqumi M., Wulff H., Skolnik E.Y. (2008). Protein histidine phosphatase 1 negatively regulates CD4 T cells by inhibiting the K^+^ channel KCa3.1. Proc. Natl. Acad. Sci. U. S. A..

[31] Panda S., Srivastava S., Li Z., Vaeth M., Fuhs S.R., Hunter T., Skolnik E.Y. (2016). Identification of PGAM5 as a mammalian protein histidine phosphatase that plays a central role to negatively regulate CD4^+^ T cells. Mol. Cell.

[32] Matsubara M., Mizuno T. (2000). The SixA phospho-histidine phosphatase modulates the ArcB phosphorelay signal transduction in *Escherichia coli*. FEBS Lett..

[33] Schulte J.E., Goulian M. (2018). The phosphohistidine phosphatase SixA targets a phosphotransferase system. mBio..

[34] Mäurer A., Wieland T., Meissl F., Niroomand F., Mehringer R., Krieglstein J., Klumpp S. (2005). The b-subunit of G proteins is a substrate of protein histidine phosphatase. Biochem. Biophys. Res. Commun..

[35] Cai X., Srivastava S., Surindran S., Li Z., Skolnik E.Y. (2014). Regulation of the epithelial Ca^2+^ channel TRPV5 by reversible histidine phosphorylation mediated by NDPK-B and PHPT1. Mol. Biol. Cell.

[36] Klumpp S., Faber D., Fischer D., Litterscheid S., Krieglstein J. (2009). Role of protein histidine phosphatase for viability of neuronal cells. Brain Res..

[37] Krieglstein J., Lehmann M., Mäurer A., Gudermann T., Pinkenburg O., Wieland T., Litterscheid S., Klumpp S. (2008). Reduced viability of neuronal cells after overexpression of protein histidine phosphatase. Neurochem. Int..

[38] Hakoshima T., Ichihara H. (2007). Structure of SixA, a histidine protein phosphatase of the ArcB histidine-containing phosphotransfer domain in *Escherichia coli*. Methods Enzymol..

[39] Hamada K., Kato M., Shimizu T., Ihara K., Mizuno T., Hakoshima T. (2005). Crystal structure of the protein histidine phosphatase SixA in the multistep His-Asp phosphorelay. Genes Cells.

[40] Rigden D.J. (2020). Protein phosphohistidine phosphatases of the HP superfamily. Methods Mol. Biol..

[41] Ogino T., Matsubara M., Kato N., Nakamura Y., Mizuno T. (1998). An *Escherichia coli* protein that exhibits phosphohistidine phosphatase activity towards the HPt domain of the ArcB sensor involved in the multistep His-Asp phosphorelay. Mol. Microbiol..

[42] Bekker M., Alexeeva S., Laan W., Sawers G., Teixeira de Mattos J., Hellingwerf K. (2010). The ArcBA two-component system of *Escherichia coli* is regulated by the redox state of both the ubiquinone and the menaquinone pool. J. Bacteriol..

[43] Pflüger-Grau K., Görke B. (2010). Regulatory roles of the bacterial nitrogen-related phosphotransferase system. Trends Microbiol..

[44] Postma P.W., Lengeler J.W., Jacobson G.R. (1993). Phosphoenolpyruvate:carbohydrate phosphotransferase systems of bacteria. Microbiol. Rev..

[45] Deutscher J., Ake F.M., Derkaoui M., Zebre A.C., Cao T.N., Bouraoui H., Kentache T., Mokhtari A., Milohanic E., Joyet P. (2014). The bacterial phosphoenolpyruvate:carbohydrate phosphotransferase system: regulation by protein phosphorylation and phosphorylation-dependent protein-protein interactions. Microbiol. Mol. Biol. Rev..

[46] Gebhardt M.J., Shuman H.A. (2017). GigA and GigB are master regulators of antibiotic resistance, stress responses, and virulence in *Acinetobacter baumannii*. J. Bacteriol..

[47] Goodwin R. (2015). Influence of Intracellular Nitrogen Status and Dynamic Control of Central Metabolism in the Plant Symbiont Sinorhizobium meliloti.

[48] Goodwin R.A., Gage D.J. (2014). Biochemical characterization of a nitrogen-type phosphotransferase system reveals that enzyme EI^Ntr^ integrates carbon and nitrogen signaling in *Sinorhizobium meliloti*. J. Bacteriol..

[49] Zimmer B., Hillmann A., Görke B. (2008). Requirements for the phosphorylation of the *Escherichia coli* EIIA^Ntr^ protein *in vivo*. FEMS Microbiol. Lett..

[50] Powell B.S., Court D.L., Inada T., Nakamura Y., Michotey V., Cui X., Reizer A., Saier M.H., Reizer J. (1995). Novel proteins of the phosphotransferase system encoded within the *rpoN* operon of *Escherichia coli*. Enzyme IIA^Ntr^ affects growth on organic nitrogen and the conditional lethality of an *era*^ts^ mutant. J. Biol. Chem..

[51] Rabus R., Reizer J., Paulsen I., Saier M.H. (1999). Enzyme I^Ntr^ from *Escherichia coli*. A novel enzyme of the phosphoenolpyruvate-dependent phosphotransferase system exhibiting strict specificity for its phosphoryl acceptor, NPr. J. Biol. Chem..

[52] Dozot M., Poncet S., Nicolas C., Copin R., Bouraoui H., Mazé A., Deutscher J., De Bolle X., Letesson J.J. (2010). Functional characterization of the incomplete phosphotransferase system (PTS) of the intracellular pathogen *Brucella melitensis*. PLoS One.

[53] Strickland M., Stanley A.M., Wang G., Botos I., Schwieters C.D., Buchanan S.K., Peterkofsky A., Tjandra N. (2016). Structure of the NPr:EIN^Ntr^ complex: mechanism for specificity in paralogous phosphotransferase systems. Structure.

[54] Li X., Peterkofsky A., Wang G. (2008). Solution structure of NPr, a bacterial signal-transducing protein that controls the phosphorylation state of the potassium transporter-regulating protein IIA^Ntr^. Amino Acids.

[55] Lasaro M., Liu Z., Bishar R., Kelly K., Chattopadhyay S., Paul S., Sokurenko E., Zhu J., Goulian M. (2014). *Escherichia coli* isolate for studying colonization of the mouse intestine and its application to two-component signaling knockouts. J. Bacteriol..

[56] Sharma R., Shimada T., Mishra V.K., Upreti S., Sardesai A.A. (2016). Growth inhibition by external potassium of *Escherichia coli* lacking PtsN (EIIA^Ntr^) is caused by potassium limitation mediated by YcgO. J. Bacteriol..

[57] Sánchez-Cañizares C., Prell J., Pini F., Rutten P., Kraxner K., Wynands B., Karunakaran R., Poole P.S. (2020). Global control of bacterial nitrogen and carbon metabolism by a PTS^Ntr^-regulated switch. Proc. Natl. Acad. Sci. U. S. A..

[58] Pflüger-Grau K., de Lorenzo V. (2014). From the phosphoenolpyruvate phosphotransferase system to selfish metabolism: a story retraced in *Pseudomonas putida*. FEMS Microbiol. Lett..

[59] Anderson B., Weigel N., Kundig W., Roseman S. (1971). Sugar transport. 3. Purification and properties of a phosphocarrier protein (HPr) of the phosphoenolpyruvate-dependent phosphotransferase system of *Escherichia coli*. J. Biol. Chem..

[60] Gassner M., Stehlik D., Schrecker O., Hengstenberg W., Maurer W., Rüterjans H. (1977). The phospho*enol*pyruvate-dependent phosphotransferase system of *Staphylococcus aureus*. 2. ^1^H and ^31^P-nuclear-magnetic-resonance studies on the phosphocarrier protein HPr, phosphohistidines and phosphorylated HPr. Eur. J. Biochem..

[61] Muimo R., Hornickova Z., Riemen C.E., Gerke V., Matthews H., Mehta A. (2000). Histidine phosphorylation of annexin I in airway epithelia. J. Biol. Chem..

[62] Wagner P.D., Vu N.D. (1995). Phosphorylation of ATP-citrate lyase by nucleoside diphosphate kinase. J. Biol. Chem..

[63] Baba T., Ara T., Hasegawa M., Takai Y., Okumura Y., Baba M., Datsenko K.A., Tomita M., Wanner B.L., Mori H. (2006). Construction of *Escherichia coli* K-12 in-frame, single-gene knockout mutants: the Keio collection. Mol. Syst. Biol..

[64] Amann E., Ochs B., Abel K.-J. (1988). Tightly regulated *tac* promoter vectors useful for the expression of unfused and fused proteins in *Escherichia coli*. Gene.

[65] Schutsky E.K., Nabel C.S., Davis A.K.F., DeNizio J.E., Kohli R.M. (2017). APOBEC3A efficiently deaminates methylated, but not TET-oxidized, cytosine bases in DNA. Nucleic Acids Res..

[66] Miller J.H. (1992). A Short Course in Bacterial Genetics.

[67] Cherepanov P.P., Wackernagel W. (1995). Gene disruption in *Escherichia coli*: Tc^R^ and Km^R^ cassettes with the option of Flp-catalyzed excision of the antibiotic-resistance determinant. Gene.

[68] Datsenko K.A., Wanner B.L. (2000). One-step inactivation of chromosomal genes in *Escherichia coli* K-12 using PCR products. Proc. Natl. Acad. Sci. U. S. A..

[69] Kumar S., Stecher G., Li M., Knyaz C., Tamura K. (2018). MEGA X: molecular evolutionary genetics analysis across computing platforms. Mol. Biol. Evol..

[70] Johnson M., Zaretskaya I., Raytselis Y., Merezhuk Y., McGinnis S., Madden T.L. (2008). NCBI BLAST: a better web interface. Nucleic Acids Res..

[71] Hall B.G. (2013). Building phylogenetic trees from molecular data with MEGA. Mol. Biol. Evol..

[72] Letunic I., Bork P. (2019). Interactive tree of life (iTOL) v4: recent updates and new developments. Nucleic Acids Res..

[73] Hu K.Y., Saier M.H. (2002). Phylogeny of phosphoryl transfer proteins of the phosphoenolpyruvate-dependent sugar-transporting phosphotransferase system. Res. Microbiol..

[74] Rasband W.S. (1997-2018). ImageJ.

