# Supplementary material for: The phosphohistidine phosphatase SixA dephosphorylates the phosphocarrier NPr
Source: J Biol Chem. 2020 Nov 23;296:100090. doi: 10.1074/jbc.RA120.015121 (PMC7948535; doi:10.1074/jbc.RA120.015121)
Supplement: Supplementary Figures and Tables [file mmc1.pdf]

Supporting information for

The phosphohistidine phosphatase SixA dephosphorylates the phosphocarrier NPr

Jane E. Schulte, Manuela Roggiani, Hui Shi, Jun Zhu, and Mark Goulian

This PDF file includes:

Supplementary Figure S1

Supplementary Figure S2

Supplementary Figure S3

Supplementary Figure S4

Supplementary Table S1

Supplementary Table S2

Supplementary Table S3

Supplementary Table S4

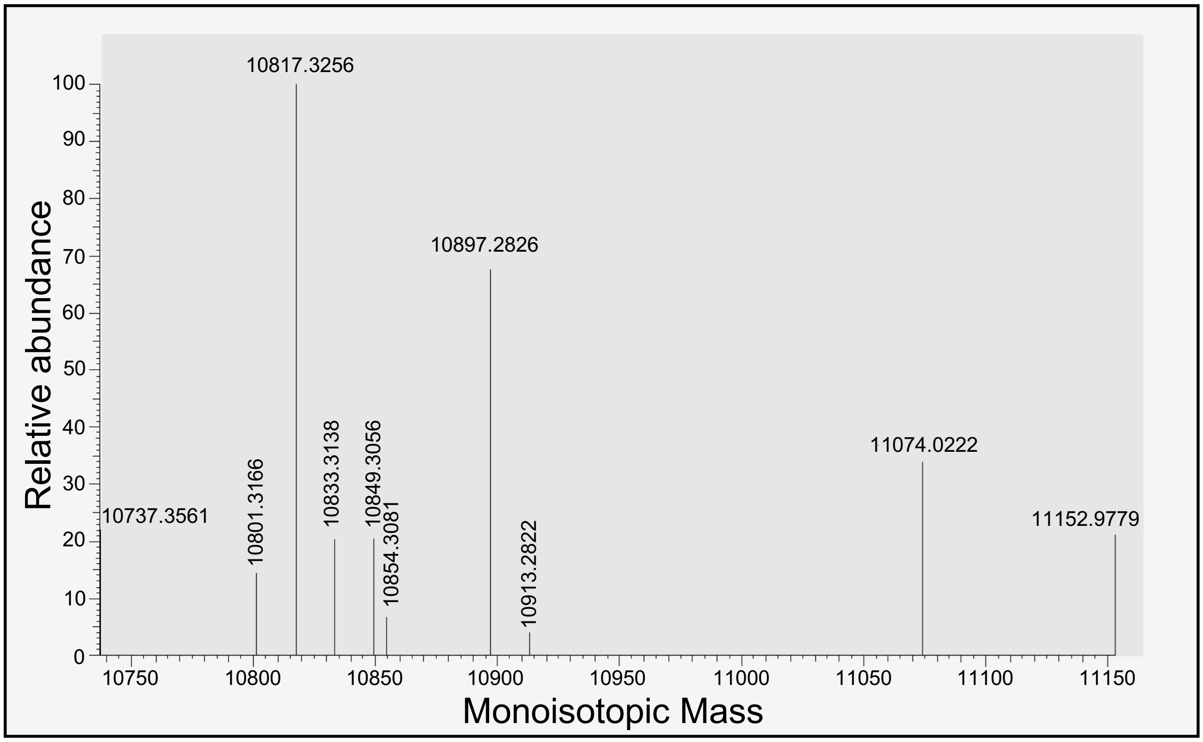

Figure S1. Analysis of a sample of phosphorylated NPr-$(His)_6$ by intact mass spectrometry suggests the presence of singly and doubly phosphorylated protein. Phosphorylated NPr-$(His)_6$ was prepared by incubating 80 μM NPr-$(His)_6$, 150 nM EI^{Ntr}, and 5 mM phosphoenolpyruvate together at 30°C for 5 h. Intact mass measurements were performed and analyzed by the Proteomics Core Facility of the Children's Hospital of Philadelphia Research Institute. The predicted monoisotopic mass for recombinant NPr-$(His)_6$ with N-terminal methionine cleavage is 10737.311 Da. The measured monoisotopic mass for mock-phosphorylated recombinant NPr-$(His)_6$ was 10737.3437. The monoisotopic mass change for phosphorylation is 80 Da.

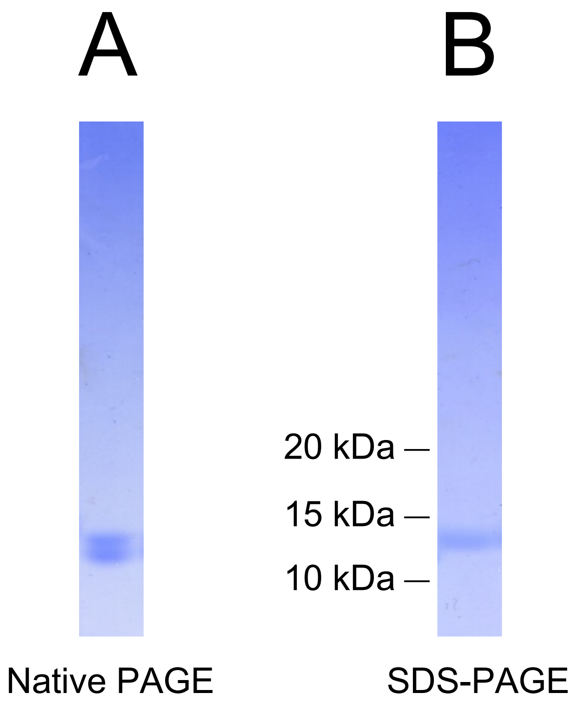

Figure S2. The NPr(H16A) doublet disappears when run under denaturing conditions. Native PAGE (A) and SDS-PAGE (B) analysis of NPr(H16A). Gels were stained with Coomassie.

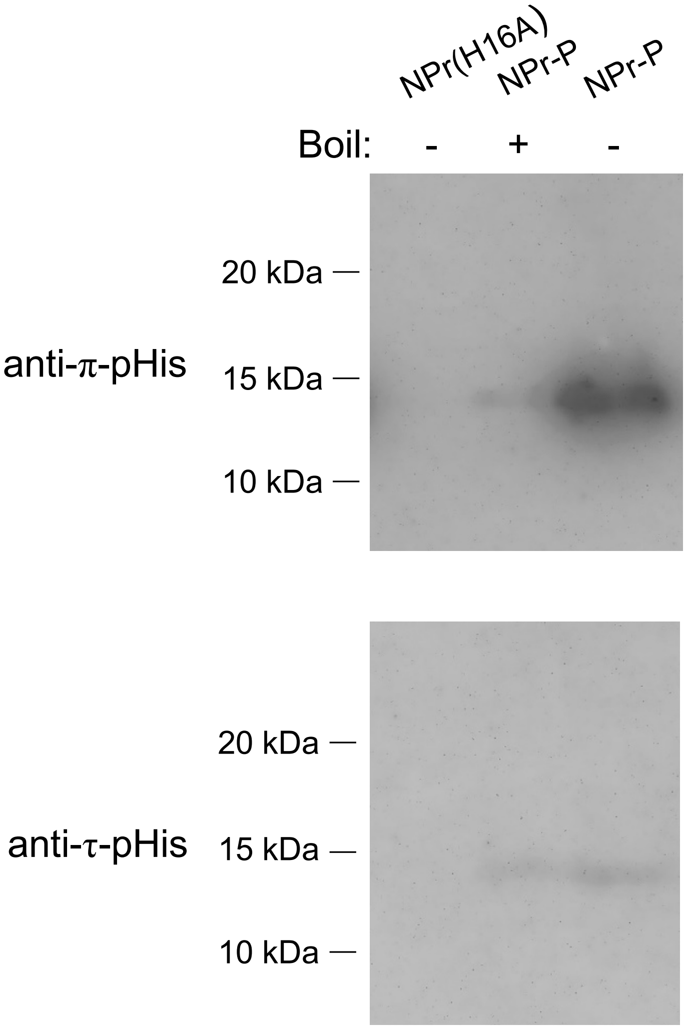

Figure S3. Faint bands observed on π-pHis and τ-pHis blots may be due to cross-reactivity with unphosphorylated His16 of NPr. Samples of NPr(H16A), which cannot be phosphorylated, and phosphorylated NPr were analyzed by SDS-PAGE and Western blot. A sample of phosphorylated NPr was prepared as described in Figure 3. Purified NPr-P was mixed with denaturing loading dye and then split into two aliquots, one of which was boiled for 10 min. The amount of protein loaded in wells for this experiment is identical to that of Figure 3 (roughly 1 μg). To better show the faint bands, the exposure time for the blots presented here is longer than the exposure time used in Figure 3.

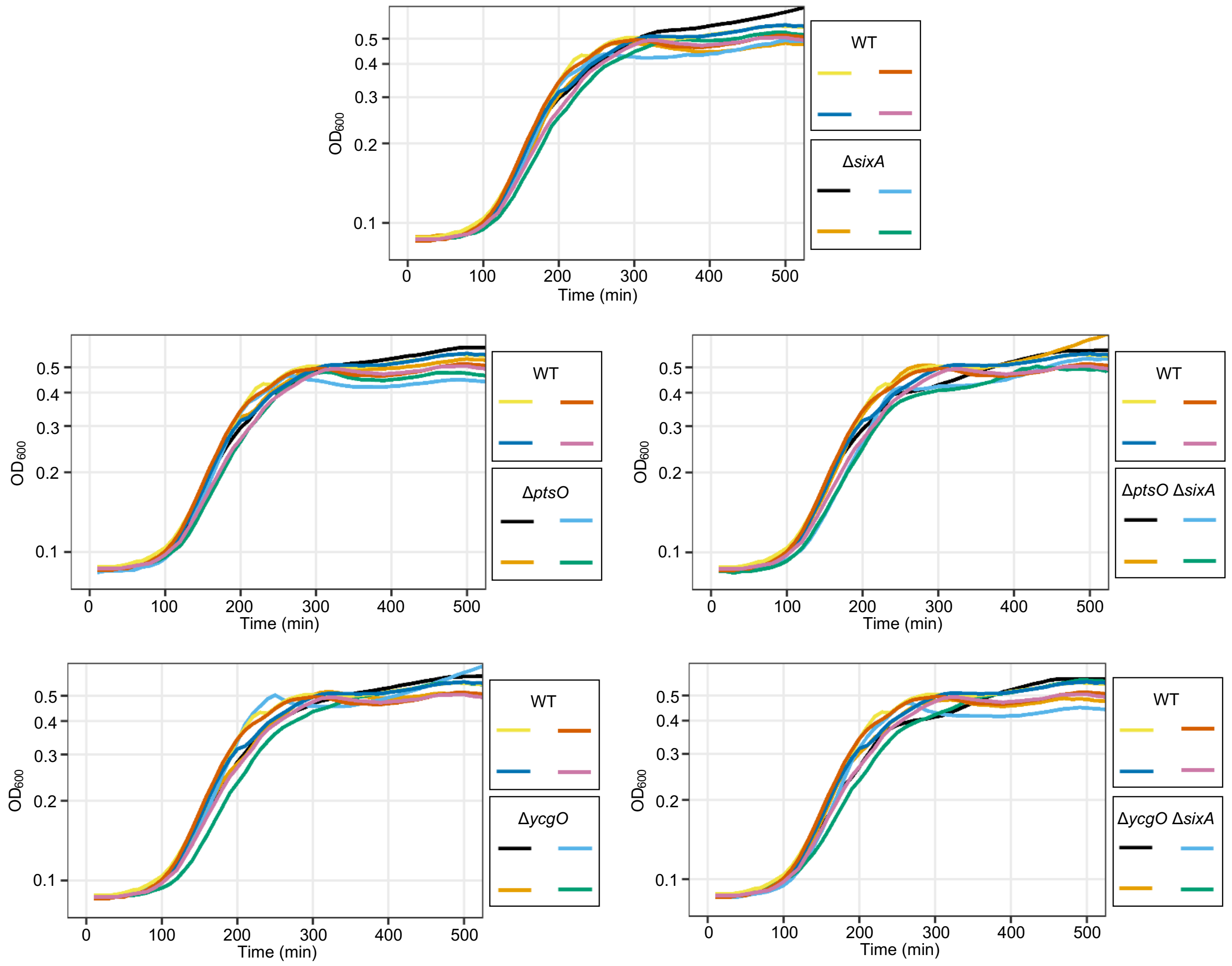

Figure S4. Growth curves for MP1 deletion strains show no growth defect relative to WT (*sixA*$^{+}$ *ptsO*$^{+}$ *ycgO*$^{+}$) in LB medium. Four biological replicates of each strain were tested by inoculating single colonies in LB medium, growing overnight at 37°C to stationary phase and then inoculating 1:1000 in 200 µl LB medium in a 96-well microplate. Optical density at 600 nm (OD_{600}) was monitored every 10 min in a BioTek® Synergy™ H1 microplate reader incubating at 37°C, with a 30 s orbital shaking period prior to each measurement. Strains used were MP7 (WT), MP204 (Δ*sixA*), MP274 (Δ*ptsO*), MP271 (Δ*ptsO* Δ*sixA*), MP275 (Δ*ycgO*) and MP273 (Δ*ycgO* Δ*sixA*).

Table S1. Bacterial strains.

**Strain**	**Relevant genotype**	**Reference or construction**
MG1655	F^- λ^- *ilvG rfb-50 rph-1*	*E. coli* Genetic Stock Center (CGSC no. 7740)
JW2337	Δ*sixA*::(FRT-*kan*-FRT)	(63)
JES13	MG1655 Δ*sixA*::(FRT)	(33)
JES208	MG1655 Δ*ptsN*::(FRT-*kan*-FRT)	(33)
JES189	MG1655 Δ*ptsP*::(FRT-*kan*-FRT)	(33)
JES190	MG1655 Δ*ptsP*::(FRT-*kan*-FRT) Δ*sixA*::(FRT)	(33)
JES286	MG1655 Δ*ptsO*::(*ptsO*-E-Tag FRT-*cat*-FRT)	MG1655 pKD46 + (*ptsO*-E-Tag FRT-*cat*-FRT)
JES287	MG1655 Δ*ptsO*::(*ptsO*-E-Tag FRT-*cat*-FRT)	$P1_{vir}$(JES286) x MG1655
JES288	MG1655 Δ*sixA*::(FRT) Δ*ptsO*::(*ptsO*-E-Tag FRT-*cat*-FRT)	$P1_{vir}$(JES286) x JES13
JES289	MG1655 Δ*ptsN*::(FRT-*kan*-FRT) Δ*ptsO*::(*ptsO*-E-Tag FRT-*cat*-FRT)	$P1_{vir}$(JES286) x JES208
JES215	MG1655 Δ*ptsP*::(FRT)	JES189 + pCP20
JES314	MG1655 Δ*ptsP*::(FRT) Δ*ptsN*::(FRT-*kan*-FRT) Δ*ptsO*::(*ptsO*-E-Tag FRT-*cat*-FRT)	$P1_{vir}$(JES289) x JES215
JES313	MG1655 Δ*ptsP*::(FRT) Δ*sixA*::(FRT)	JES190 + pCP20
JES315	MG1655 ΔptsP::(FRT) ΔsixA::(FRT) Δ*ptsN*::(FRT-*kan*-FRT) Δ*ptsO*::(*ptsO*-E-Tag FRT-*cat*-FRT)	$P1_{vir}$(JES289) x JES313
MP1		(55)
MP203	MP1 Δ*sixA*::(FRT-*kan*-FRT)	MP1 pKD46 + (FRT-*kan*-FRT)
MP216	MP1 Δ*sixA*::(FRT-*kan*-FRT)	$P1_{vir}$(MP203) x MP1
MP235	MP1 Δ*sixA*::(FRT)	MP216 + pCP20
MP7	MP1 attλ::(*cat tetR tetA-mcherry*)	(55)
MP13	MP1 attλ::(*cat tetR tetA-gfpmut3.1*)	(55)
MP204	MP1 Δ*sixA*::(FRT-*kan*-FRT) attλ::(*cat tetR tetA-gfpmut3.1*)	$P1_{vir}$(MP203) x MP13
MP259	MP1 Δ*sixA*::(FRT) attλ::(*cat tetR tetA-gfpmut3.1*)	MP204 + pCP20
MP263	MP1 Δ*ptsO*::(FRT-*kan*-FRT)	MP1 pKD46 + (FRT-*kan*-FRT)
MP270	MP1 Δ*ptsO*::(FRT-*kan*-FRT) attλ::(*cat tetR tetA-mcherry*)	$P1_{vir}$(MP263) x MP7
MP274	MP1 Δ*ptsO*::(FRT) attλ::(*cat tetR tetA-mcherry*)	MP270 + pCP20
MP271	MP1 Δ*sixA*::(FRT) Δ*ptsO*::(FRT-*kan*-FRT) attλ::(*cat tetR tetA-gfpmut3.1*)	$P1_{vir}$(MP263) x MP259
MP264	MP1 Δ*ycgO*::(FRT-*kan*-FRT)	MP1 pKD46 + (FRT-*kan*-FRT)
MP272	MP1 Δ*ycgO*::(FRT-*kan*-FRT) attλ::(*cat tetR tetA-mcherry*)	$P1_{vir}$(MP264) x MP7
MP275	MP1 Δ*ycgO*::(FRT) attλ::(*cat tetR tetA-mcherry*)	MP272 + pCP20
MP273	MP1 Δ*sixA*::(FRT) Δ*ycgO*::(FRT-*kan*-FRT) attλ::(*cat tetR tetA-gfpmut3.1*)	$P1_{vir}$(MP264) x MP259
TOP10	F^- *mcrA* Δ(*mrr-hsdRMS-mcrBC*) *endA1 recA1* φ80*lacZ*ΔM15 Δ*lacX74 araD139* Δ(*ara-leu*)*7697 galU galK rpsL nupG*	Invitrogen
BL21(DE3)	F^- *ompT* $hsdS_B$ (r_B^- m_B^-) *gal dcm* (DE3)	Novagen

Table S2. Plasmids.

Plasmid	Relevant features	Reference
pTrc99a	*lacI*^{q}, P$_{trc}$, MCS from pUC18, *rrnB*(Ter), *bla*, ori pMB1	(64)
pSixA (pJS17)	pTrc99a P$_{trc}$-*sixA*	(33)
pSixA(H8A) (pJS21)	pTrc99a P$_{trc}$-*sixA*(H8A)	(33)
pET-22b(+)	*lacI*, P$_{T7lac}$, MCS, C-terminal (His)$_{6}$-tag, *bla*	Novagen
pJS43	pET-22b(+) P$_{T7lac}$-*ptsO*-(His)$_{6}$	This study
pJS47	pET-22b(+) P$_{T7lac}$-*ptsO*(H16A)-(His)$_{6}$	This study
pET-41	*lacI*, P$_{T7lac}$, *kan*	Novagen
pEKS001	pET-41 P$_{T7lac}$-MBP-TEV-*A3A*-TEV-(His)$_{8}$	(65)
pJS65	pET-41 P$_{T7lac}$-*ptsO*-TEV-(His)$_{8}$	This study
pJS69	pET-41 P$_{T7lac}$-*ptsO*(H16A)-TEV-(His)$_{8}$	This study
pJS67	pET-41 P$_{T7lac}$-*sixA*-TEV-(His)$_{8}$	This study
pJS70	pET-41 P$_{T7lac}$-*sixA*(H8A)-TEV-(His)$_{8}$	This study
pCP20	λ*c*I857(ts), λ*p*$_{R}$-FLP, *repA101*(ts), *oriR101*, *bla*, *cat*	(67)
pKD3	*ori*R6Kγ, *bla*, FRT-*cat*-FRT	(68)
pKD46	*repA101*(ts), *oriR101*, *bla*, P$_{araB}$-(*gam bet exo*)	(68)
pKD13	*ori*R6Kγ, *bla*, FRT-*kan*-FRT	(68)

Table S3. Oligonucleotide primers.

Primer	Sequence	Resulting construct or purpose
npr-Etag-lred-F	GGCCGCCGTTATCGCCCTCTTTAATTCTGGTTTTGATGAAGATGGAGCGCCAGTTCCGTACCCAGACCCGTTAGAACCTCGTTAAATGGTCCATATGAATATCCTCCTTAGTTCC	*ptsO*-E-Tag-FRT-*cat*-FRT for tagged NPr strain (JES286)
npr-Etag-lred-R	TGATTTTCTTATTGTCGGGGGGAGTTTGAAGGGAGTTGTATGTCAAAGTGCTTGAGCGATTGTGTAGGCTGG	*ptsO*-E-Tag-FRT-*cat*-FRT for tagged NPr strain (JES286)
sixA-del-U1	GGAAAACAACTGCAACTGACCTGCAATAAG	FRT-*kan*-FRT for MP1 Δ*sixA* (MP203)
sixA-del-L1	GGAGAAACTGCCCGGTGTACGCGCACGTAACCGGGCATTGATTGCTTAAATTGCTTTTGCCATCTTTGTAGG	FRT-*kan*-FRT for MP1 Δ*sixA* (MP203)
MP1-npr-lred-u	GGTAAAAACGTCCAGTCACGCCATCGGACGCTGGAAAAACGTAAACCATGATTCCGGGGATCCGTCGACC	FRT-*kan*-FRT for MP1 Δ*ptsO* (MP263)
MP1-npr-lred-d2	CAGTACGCGCTTTTCTTATTGCCGGGGGAGCTTGAAGGGATTTGAATGTCAAAGTGATGAAGATTAATCTTCATCAAAACCAGATGTAGGCTGGAGCTGCTTCG	FRT-*kan*-FRT for MP1 Δ*ptsO* (MP263)
MP1-ycgO-lred-u	ATGGTAGGGTTTATATAAGAGACAGCGTAATCAGGAGTAACCGACCGTTGATTCCGGGGATCCGTCGACC	FRT-*kan*-FRT for MP1 Δ*ycgO* (MP264)
MP1-ycgO-lred-d2	GGTGTAACTTGTTATCGCTGGATGCGACCAGCGTCGTATCCAGCGTTATAAAACACATTAAACTTAAGATTCGGCTTCTTCCTCTGTAGGCTGGAGCTGCTTCG	FRT-*kan*-FRT for MP1 Δ*ycgO* (MP264)
NdeI-ptsO-F	AACGTAACATATGACCGTCAAGCAAACTGT	pJS43
XhoI-ptsO-R	GCATGTCTCGAGATCTTCATCAAAACC	pJS43
npr-H16A-F	CATGCCCAGCTTGTTTGTG	pJS47
npr-H16A-R	GCGGCCCGGCCTGCAATG	pJS47
ptsO-TEV-vec	ATTCTGGTTTTGATGAAGATGGTACCGAAAACCTGTATTTTCAGGGC	pJS65
ptsO-pEKS-vec	ACAGTTTGCTTGACGGTCATATGTATATCTCCTTCTTAAAG	pJS65
ptsO-pEKS-ins	TTTAAGAAGGAGATATACATATGACCGTCAAGCAAACTGTTG	pJS65
ptsO-TEV-ins	AAATACAGGTTTTCGGTACCATCTTCATCAAAACCAGAAT	pJS65
pBR322-seq-Rev	GCGATATAGGCGCCAGCAAC	pJS69, pJS70
NPr-H16A-R1	CAGGCCGGGCCGCCATGCCCAG	pJS69
NPr-H16A-F2	TGGGCATGGCGGCCCGGCCTG	pJS69
pET-seq-F	CCTCAAGACCCGTTTAGAGG	pJS69, pJS70
pBR322-seq-Rev-comp	GTTGCTGGCGCCTATATCGC	pJS69, pJS70

pET-seq-F-comp	CCTCTAAACGGGTCTTGAGG	pJS69, pJS70
sixA-TEV-vec	TGAAGATGGCAAAAGCTATCGGTACCGAAAACCT GTATTTTCAGGGC	pJS67
sixA-pEKS-vec	GACGCATGATAAAAACTTGCATATGTATATCTCC TTCTTAAAG	pJS67
sixA-pEKS-ins	TTTAAGAAGGAGATATACATATGCAAGTTTTTAT CATGCGTC	pJS67
sixA-TEV-ins	GCCCTGAAAATACAGGTTTTCGGTACCGATAGCT TTTGCCATCTTCAG	pJS67
SixA-H8A-R1	CTGCGTCGCCCGCACGCATG	pJS70
SixA-H8A-F2	CATGCGTGCGGGCGACGCAG	pJS70

Table S4. SixA and NPr homolog NCBI accession information.

Species	Taxonomy ID	SixA accession	NPr accession
Acidovorax konjaci	32040	WP_092950243	WP_092957584
Acinetobacter baumannii	470	WP_034704059	WP_088757932
Agrobacterium tumefaciens	358	WP_012651600	WP_003505372
Arcobacter cryaerophilus	28198	WP_066157277	-
Bdellovibrio bacteriovorus	959	WP_061836576	-
Bradyrhizobium yuanmingense	108015	WP_085969295	WP_050991904
Brevundimonas diminuta	293	WP_088410353	WP_040344675
Brucella abortus	235	WP_057056868	WP_002965161
Burkholderiales bacterium	1891238	WP_105479956	WP_105480856
Campylobacter concisus	199	WP_149719497	-
Cetia pacifica	1424653	WP_123352084	-
Chondromyces crocatus	52	WP_050430834	WP_050431271
Citrobacter braakii	57706	WP_131405187	WP_075205750
Corallococcus exiguus	83462	WP_120559234	WP_120561227
Coxiella burnetii	227377	NP_819151	NP_819769
Enhygromyxa salina	215803	WP_106092305	WP_106092972
Escherichia coli	511145	NP_416842	NP_417673
Geoalkalibacter subterraneus	483547	WP_052464416	WP_040200827
Haemophilus influenzae	727	WP_110431846	WP_163470595
Helicobacter japonicus	425400	WP_034362869	-
Hydrogenimonas thermophila	223786	WP_092913790	-
Klebsiella pneumoniae	573	WP_117681860	WP_159188967
Labilithrix luteola	1391654	WP_146647283	WP_146653493
Legionella pneumophila	446	WP_029346073	WP_010946224
Limnohabitans curvus	323423	WP_108358906	WP_108360284
Mesorhizobium tianshanense	39844	WP_145720490	WP_145720571
Methylophilus rhizosphaerae	492660	WP_091471879	WP_091472105
Minicystis rosea	888845	WP_146732274	WP_146728355
Myxococcus fulvus	33	WP_046715324	WP_046716353
Paucimonas lemoignei	29443	WP_132257534	WP_132257963
Polyangium fumosum	889272	WP_136931974	WP_136935021
Pseudomonas aeruginosa	208964	NP_250307	NP_253156
Rhizobium leguminosarum	384	WP_168325921	WP_018068902
Rhodoferax koreense	1842727	WP_076199826	WP_076196429
Salmonella enterica	28901	WP_079974159	WP_001582734
Sinorhizobium saheli	36856	WP_066868574	WP_153435426
Stigmatella erecta	83460	WP_093517267	WP_075009929
Sulfurospirillum cavolei	366522	WP_060825823	-
Thermodesulforhabdus norvegica	39841	WP_143083081	WP_093396362
Vibrio cholerae	666	WP_076025212	WP_148518799
Vulgatibacter incomptus	1391653	WP_050724250	WP_050726426
Xanthomonas albilineans	29447	WP_045766856	WP_012916628

Zoogloea oleivorans	1552750	WP_148580229	WP_148580781
